# Artificial intelligence enhances genomic surveillance in healthcare outbreak investigations

**DOI:** 10.1017/ice.2025.10355

**Published:** 2026-02

**Authors:** Alexander Sundermann, Jieshi Chen, Melissa Saul, Kathleen Shutt, Marissa Griffith, Graham Snyder, Lora Lee Pless, Artur Dubrawski, Lee Harrison

**Affiliations:** 1 Microbial Genomic Epidemiology Laboratory, Center for Genomic Epidemiology, University of Pittsburgh, Pittsburgh, PA, USA; 2 Division of Infectious Diseases, University of Pittsburgh School of Medicine, Pittsburgh, PA, USA; 3 Department of Epidemiology, School of Public Health, University of Pittsburgh, Pittsburgh, PA, USA; 4 Auton Lab, Carnegie Mellon University, Pittsburgh, PA, USA; 5 Department of Medicine, University of Pittsburgh, Pittsburgh, PA, USA; 6 Department of Infection Control and Hospital Epidemiology, UPMC Presbyterian, Pittsburgh, PA, USA

## Abstract

**Background:**

Outbreak investigation and control are critical for preventing the spread of infectious diseases in healthcare settings. Traditional methods rely on manual processes, which are time-consuming and limited in scope. Whole genome sequencing (WGS) surveillance improves outbreak detection but still requires extensive manual chart reviews to identify transmission routes. Integrating artificial intelligence (AI) may enhance the efficiency and accuracy of these investigations.

**Methods:**

We evaluated an AI tool developed to streamline healthcare outbreak investigations detected by the Enhanced Detection System for Healthcare-associated Transmission (EDS-HAT). For outbreaks detected between November 2021 and November 2023, multiple data elements were extracted from electronic health records (EHR) for all patients. The AI algorithm was applied to identify transmission routes, and its performance was assessed against expert manual reviews. Key measures included additional transmission routes identified and sensitivity.

**Results:**

Data from 172 outbreaks involving 476 case patients were analyzed. The AI tool identified 37 transmission routes that were missed by manual review, including procedures and provider routes. The algorithm achieved a sensitivity of 76.0% (95% confidence interval [CI] 71.1%–81.1%) compared to manual review, increasing to 91.7% (95% CI 87.7%–94.7%) after accounting for transmission at other facilities and downstream exposures.

**Conclusion:**

The EDS-HAT AI tool significantly improved outbreak investigations by automating the identification of transmission routes, both with concordant findings of manual review as well as finding additional routes of transmission missed by traditional chart review. AI with genomic surveillance has the potential to optimize outbreak detection and investigation to streamline interventions in healthcare settings.

## Introduction

Outbreak detection, investigation, and control are fundamental components to mitigate the spread of infectious diseases in hospitals. Traditional methods of identifying suspected transmission routes during outbreak investigations, which is necessary for the development of interventions to interrupt transmission, rely on manual data review by infection preventionists (IPs) and hospital epidemiologists.^1^ Each outbreak requires a rigorous chart review of shared patient exposures to direct interventions. This approach can be inaccurate and is limited by the human capacity to analyze large, complex data sets. Moreover, this process is time-consuming and can fail to identify the responsible transmission route, potentially allowing an outbreak to spread and cause additional serious, potentially preventable infections.

The emerging approach of whole genome sequencing (WGS) surveillance provides more accurate outbreak detection compared to traditional methods.^2^ WGS enables the precise identification of genetic similarities between pathogens, thereby facilitating the detection of transmission events that could otherwise go unnoticed. While this approach identifies the occurrence of an outbreak, an extensive chart review is still needed to identify a suspected transmission route; this remains a substantial bottleneck in outbreak investigations.^3^ In fact, as WGS surveillance identifies more outbreaks, the time required to investigate these outbreaks can substantially increase, further straining resources and delaying interventions.

To address these challenges, integrating advanced technologies such as artificial intelligence (AI) into outbreak investigation processes has shown significant promise.^4^ In 2016, we developed the Enhanced Detection System for Healthcare-Associated Transmission (EDS-HAT), a novel application that leverages AI to enhance and streamline the identification of transmission routes for outbreaks detected by WGS surveillance.^5–8^ EDS-HAT aims to improve the speed, accuracy, and comprehensiveness of outbreak detection by automating the analysis of electronic health records (EHRs) and combining this with accurate outbreak data from WGS surveillance. Our analysis during the creation and validation of this tool showed promise in accurately and quickly identifying outbreak transmission routes.^7^

This study aims to evaluate the performance of the EDS-HAT AI tool in enhancing outbreak investigation. Specifically, we assessed its performance over a 2-year period during which real-time WGS surveillance and infection prevention and control (IP&C) interventions were conducted.^9^ While a preliminary analysis of EDS-HAT’s AI potential has been previously reported,^7^ the present study expands on that work by applying the algorithm to a larger and more diverse cohort,^9^ evaluating additional outcomes, and exploring the tool’s use cases in greater depth. These extensions provide a more comprehensive assessment of EDS-HAT’s performance and its potential value for real-world infection prevention programs. Additionally, unlike our previous work, this study directly compares the AI algorithm’s findings against traditional infection prevention investigations, allowing for a more rigorous evaluation of its utility.

## Methods

### Study setting

This study was performed at the UPMC Presbyterian hospital, an adult tertiary care hospital with 694 total beds, 134 critical care beds, and over 400 annual solid organ transplantations. Ethics approval was obtained from the University of Pittsburgh Institutional Review Board.

### WGS Surveillance

We used real-time genomic surveillance to identify outbreaks among inpatients at UPMC caused by select bacterial pathogens between November 2021 and November 2023 which is reported separately.^9^ Isolates were sequenced weekly to identify outbreaks. Investigators performed EHR review on patients involved in the outbreak to identify common patient exposures. When a plausible transmission route was identified, we informed the IP&C department, who conducted a secondary review and then implemented interventions to prevent additional infections.

### EHR Data Extraction and Processing

**Figure 1.**
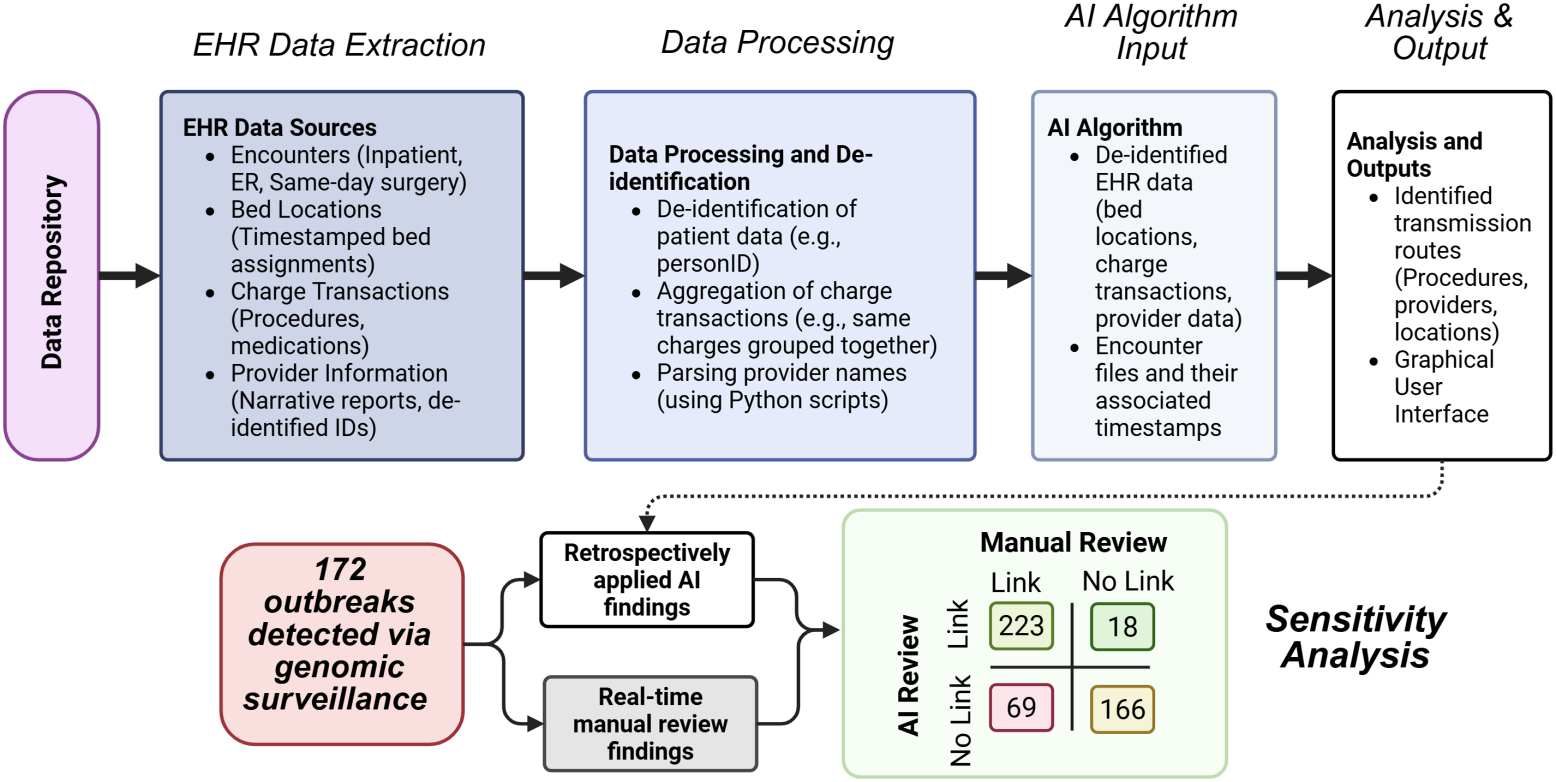
Data flow and comparative analysis of artificial intelligence-enhanced outbreak review versus manual review.

We included all inpatient, emergency room (ER), and same-day surgery patients admitted or visiting the hospital between November 1, 2021, and November 1, 2023. We extracted a subset of the UPMC EHR for these patients 120 days prior to each admission date through the discharge date (Figure 1). The data repository contained detailed records of admissions, discharges, and transfers across UPMC hospitals and clinics. Variables obtained included patient account numbers, hospital locations, patient type (inpatient, ER, or same-day surgery), medical record numbers (MRN), admission dates, and discharge dates. A unique identifier per patient, termed “acctid,” was generated from a substring of the hospital account number. The “acctid” remains consistent across multiple visits, enabling longitudinal tracking of patient encounters.

For bed location data, we queried the admissions/discharges/transfers database to track each patient’s assigned bed during their hospital stay. Each location was timestamped, detailing the start and end times for the patient’s bed assignment. The extracted data also included the patient acctid, hospital location, unit, room, and bed number, providing precise information about patient movement.

Additionally, we extracted charge transactions from the financial database and clinical reports from the EHR that included history and physical reports, progress notes, discharge summaries, and consult notes. Charge transactions were matched to patient encounters, which included date of service, department, transaction code, and quantity; thus, providing insight into services rendered to each patient during each stay. These transactions were summarized by acctid, service date, and charge transaction code to remove zero quantities and aggregate charges per day. Code groupings were created based on the location of the transaction code (eg, all codes in the radiology suite or cardiac catheterization lab) or aggregated codes representing a single exposure (eg, multiple codes for endoscopy mapped to a single group charge code or various doses of a medication mapped to a single medication). From the clinical reports we used a customized Python script to identify providers with a regular list of expressions, of which is possible at our healthcare center since our EHR uses templates for inpatient notes. This script was trained to identify and de-identify providers, generating a unique provider ID for each instance where a provider name was in the report. This process provides anonymity of healthcare workers while retaining the ability to map their involvement in patient care.

Moreover, patient identities from the extracted data were de-identified using the De-ID program (De-ID Data, Philadelphia, PA); each patient acctid was utilized to generate reproducible identifiers for patients (personID). Linkage files were generated to allow re-identification, when necessary, for the IP&C team. The de-identified data files were then compressed and securely uploaded to a University of Pittsburgh OneDrive, where they were linked into the AI algorithm for further analysis.

### Artificial Intelligence Algorithm

The AI algorithm employed in this study is based on our previously published algorithm, which uses Bayesian inference for model parameters and incorporates case-control methodology, an approach often employed by epidemiologists for identifying outbreak transmission routes.^10^ Case patients were defined as those with clinical isolates that clustered by WGS, as described above.^5,8^ Control patients included all individuals hospitalized in the 30 days prior to a case patient’s culture date who did not have a positive result for the genetically related strain. Analyzes were performed using either all other hospital patients as controls or hospital unit-based controls. Only route exposures on or before a case patient’s culture date were considered. The algorithm was applied at the conclusion of the two-year study period to investigate the potential benefit of AI for identifying transmission routes.

The AI algorithm evaluates each outbreak by calculating the maximum log-likelihood ratio of observing the case infections, given that exposure to the principal transmission route probabilistically causes infection, compared to the likelihood of a non-transmission explanation.^8^ A constant patient-to-patient transmission likelihood is added for each case infection not exposed to the principal transmission route. Routes were categorized by the type of transmission route such as procedure, healthcare worker, or location.

A graphical user interface was created to present each outbreak’s results with relevant information for rapid investigator review such as algorithm rank, case patients exposed to the route, control patients exposed to a route, odds ratios, and other relevant epidemiological information. Top routes were identified as those with the numerically highest odds ratios. Combined with the AI results were visual graphics displaying bed locations, visuals of the charge code exposure dates relative to the positive culture date of case patients, and the ability to review results as the outbreak progressed (eg, at 2 case patients, then 3 case patients, etc.).

### AI Performance

The AI algorithm was applied retrospectively on all investigated outbreaks during the study period and output examined at each incremental additional patient within an outbreak (Figure 1). The algorithm was evaluated based on its ability to find concordant results with manual expert investigations, as well as to identify additional, previously missed routes of transmission within outbreaks. For discordant results, where a transmission route was identified manually but not detected by the AI algorithm, an in-depth review was performed to determine potential causes of discordance. These findings were then summarized to provide insights into the algorithm’s performance and areas for improvement. Additionally, the sensitivity of the AI algorithm, defined as its ability to find an epidemiological link for a patient with one identified manually, was calculated.

## Results

### EHR Data

Our database included 48,723 patients. There were 13,503 healthcare workers associated with care of these patients, 224 department codes, and 14,938 individual transaction codes; these individual transaction codes were further grouped into 137 categories (range: 2–1,818 codes per group; average: 109.8 codes per group).

### AI Performance and Characteristics

**Figure 2.**
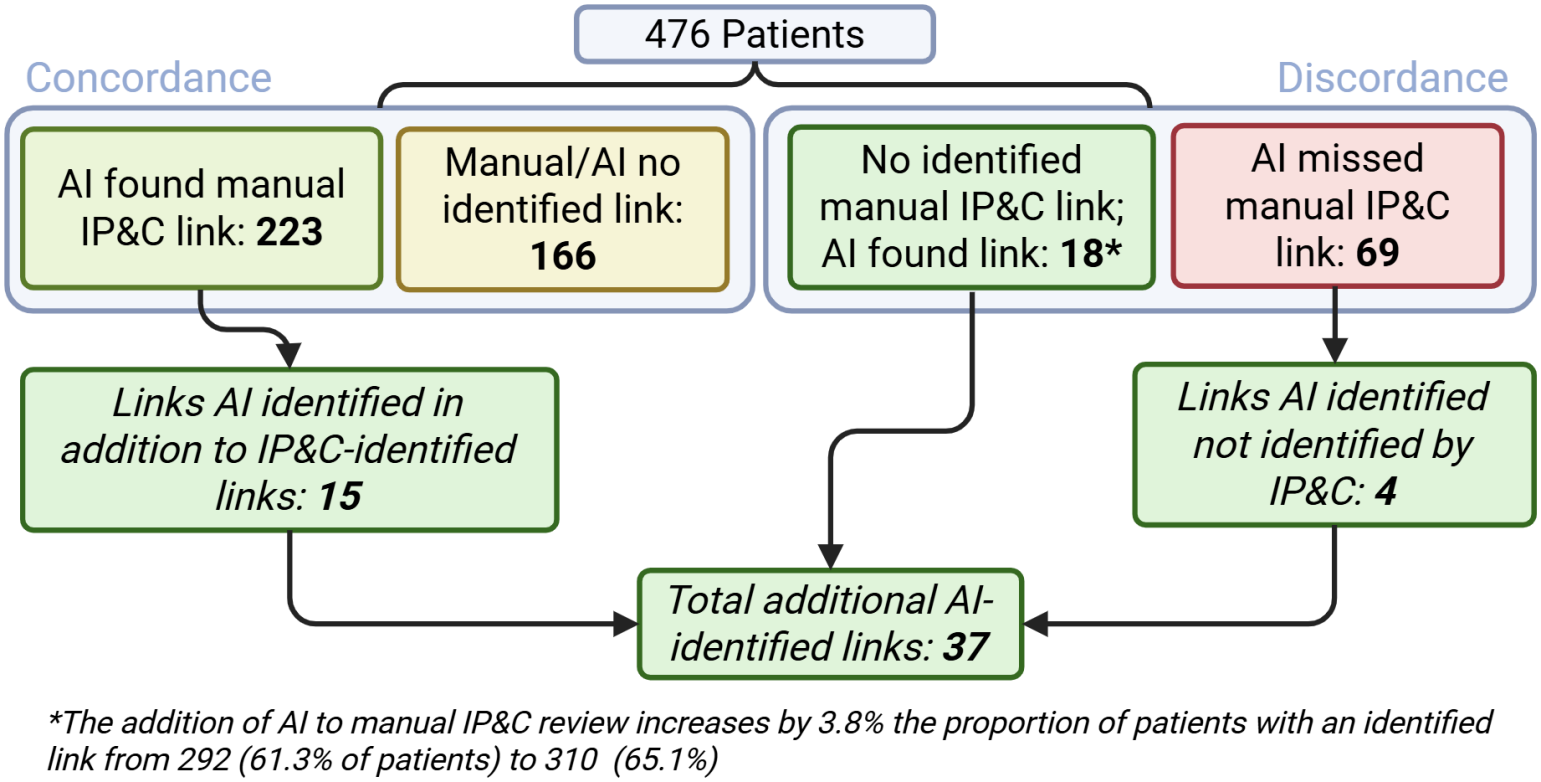
Comparison of manual and artificial intelligence-identified transmission links among outbreak patients.

There were 172 WGS-defined outbreaks involving 476 patients (2-16 patients per outbreak), of which 292 (61.3%) had a transmission route that was identified manually.^9^ The AI algorithm was concordant with 76.4% (223/292) patient isolates that had a manually identified transmission route and found 15 additional, potential transmission routes among the 223 patients (Figure 2). Neither the AI algorithm nor manual IP&C review identified any transmission route for 34% (166/476) infections. However, there were 3.8% (18/476) patient infections for which no manual IP&C route was identified, but the AI algorithm identified plausible routes of transmission that were otherwise missed. The AI algorithm missed routes identified by manual investigation in 14.5% (69/476) patient infections but did find 4 alternative routes of transmission within this category. Overall, the use of the AI algorithm had the potential to increase the identification of epidemiological links by 3.8% (18 links; from 61.3% to 65.1%).

### Nature of AI Insights

Of the 37 transmission routes that were detected by the AI algorithm but not manual review, 16 (43.2%) were procedures across four outbreaks, including echocardiograms, interventional radiology procedures, and continuous renal replacement therapy. There were 14 (37.8%) new provider routes identified within five outbreaks. One notable example was an outbreak of *Klebsiella pneumoniae* among five patients housed on different units. The AI algorithm noted a single provider providing care to all patients before their infections, suggesting possible transmission via contaminated healthcare worker hands, attire, or reusable medical equipment. Lastly, 7 (18.9%) routes were identified as inpatient units that had not previously been detected, for example, these routes were missed by traditional, manual review of the EHR.

### Analysis of transmission routes missed by AI

The 69 missed transmission routes could be categorized into four issues (Table 1). First, there were 27 (39.1%) patients admitted from outside facilities where WGS was performed for an infection that was present upon admission. Manual review identified the outside facility as the only commonality among the outbreaks and patients; however, there are no charge codes or admission codes for these facilities. In addition, EDS-HAT was designed to only detect transmission at our hospital and not other facilities. Second, there were 18 (26.1%) infections in which the determined route of transmission did not have an associated charge code. Third, for 4 (5.8%) cases, the transmission route was not highly ranked by the algorithm. Lastly, 20 (28.9%) infections involved scenarios where the source patient, after developing an infection, subsequently contaminated the transmission route, thus exposing the next patient. The algorithm is constrained by the assumption that a single exposure must precede both infections and is therefore unable to identify scenarios where an infected patient subsequently contaminated a transmission route. For example, a patient was admitted to an intensive care unit and developed an infection. The next patient was then admitted to the same unit, next to the first patient, and subsequently developed the same infection with the same strain.

**Table 1.** Missed routes of transmission by the artificial intelligence (AI) algorithm that had been identified by manual review

Issue identified	Count (%)	Reasoning
Outside facility	27 (39.1%)	Outside facilities did not have charge codes representing admission from their facility
Post-positive scenario	20 (28.9%)	AI algorithm assumes both patients exposures occur prior to their infection rather than the initial patient “contaminating” a route that is then exposed to a second patient
Missing charge	18 (26.1%)	Charge code does not exist within the patient record
Missed route	4 (5.8%)	AI algorithm did not rank any transmission route high

### Performance of AI

The overall sensitivity of the AI algorithm to identify manual links was 76.4% (223 concordant results and 69 discordant results; 95% confidence interval [CI] 71.1%–81.1%). Excluding the outside facility scenario (27 patients), the sensitivity improved to 84.2% (42 discordant results; 95% CI 79.2%–88.3%). Further excluding the post-positive scenario (20 patients), the sensitivity increased to 91.7% (22 discordant results; 95% CI 87.7%–94.7%).

## Discussion

In this study, we found that our EDS-HAT AI tool agreed with manual chart review findings with an overall sensitivity of 76.4%, which increased to 91.7% when adjusted for specific scenarios. We also determined that the tool can be a highly useful adjunct for this purpose in outbreak investigations because it identified transmission routes in 3.8% of cases that were otherwise missed by traditional review methods and occurred more quickly. While the absolute increase in identified transmission routes was modest, the ability of the AI tool to automate and scale this process suggests that it may serve as an important and useful tool for initial review of a transmission event, allowing IP&C teams to reallocate time toward intervention and response. Its value lies not only in detecting missed routes but also in potential reduction in time spent on repetitive review tasks and standardizing outbreak investigations.

Our EDS-HAT AI tool has shown promise in prior publications for detecting transmission routes in various applications.^5–7^ We previously demonstrated its ability to detect major outbreak transmission routes that are often considered nontraditional, as outbreaks are usually investigated based on spatial and temporal commonalities.

IP&C teams typically spend approximately 25% of their time on surveillance and outbreak investigation.^12^ WGS surveillance has been shown to significantly improve outbreak detection by identifying outbreaks that would otherwise be missed.^2,3^ This has raised concerns about the increased time required to review and intervene in these outbreaks. Here, we provide a possible solution to the increased time burden that results from the higher volume of outbreaks detected through genomic surveillance. By automating a major portion of the review process, the AI tool can help IP&C teams focus their time on actionable transmission routes both by identifying missed links and by deprioritizing clusters where neither manual review nor the AI find plausible connections, thereby improving overall efficiency.

Although our long-term goal is real-time integration of the AI algorithm, current implementation of the AI algorithm occurs with a time lag of approximately one month, as data extraction, processing, and review are performed retrospectively after we have already received and intervened upon real-time genomic surveillance. Key challenges to achieving true real-time functionality include ensuring compatibility with hospital information systems, optimizing automated data workflows, and maintaining data security. Other health systems looking to adopt a similar system may encounter similar difficulties; however, our experience demonstrates that creating such a system is possible.

Similar to any diagnostic medical test, it is important that AI algorithms be useful to make an effective change. Our goal was to enhance outbreak investigation with WGS surveillance to prevent additional infections. Moreover, we must consider the end users of this program: IP&C teams. IPs are critical personnel within the healthcare setting and must be knowledgeable about the uses and applications of this tool. Our research team worked closely with our IP&C team over multiple years to facilitate the adoption of EDS-HAT. Other institutions should ensure the presence of proper capabilities and capacity to implement such systems effectively. One possible approach, which we are developing, is a user interface that allows for rapid review and visualizations.

There are limitations to our study. First, our AI algorithm was unable to detect routes of transmission for 166 infections where manual review also did not find transmission routes. However, our prior findings suggest that some infections may have been misclassified as part of an outbreak due to an overly permissive single nucleotide polymorphism (SNP) threshold for defining relatedness.^9^ To address this, we subsequently lowered our threshold for pairwise SNP differences from 15 to 10. The AI algorithm supports this approach by similarly failing to identify possible transmission routes for these cases. Second, our analysis was conducted at a large tertiary care center, a setting that is not generalizable for all types of healthcare institutions. Third, our algorithm was limited to exposures recorded in charge codes and clinicians documenting in the patient chart. While we encountered instances of missing charge codes, these occurrences were rare. Fourth, our genomic surveillance relied solely on clinical isolates and did not include asymptomatic screening or colonization cultures. As a result, transmission events involving colonized but undiagnosed individuals may have been missed, potentially limiting the algorithm’s ability to detect all transmission routes.

Following this analysis, what is the role of an AI algorithm in hospital WGS surveillance? The algorithm serves as a valuable adjunct to IPs, helping to identify likely transmission routes by analyzing patterns in complex EHR data after genomic clustering has been established. By rapidly scanning for overlaps in patient locations, medical procedures, staff interactions, and other exposures, the model can prioritize plausible transmission routes for further investigation. However, our findings also underscore the algorithm’s current limitations particularly in complex scenarios such as post-infection route contamination. These cases were missed by the AI model and only identified through expert manual review. This highlights that while this approach may improve the speed and scale of genomic surveillance, expert oversight remains essential for identifying complex transmission events, especially those involving indirect transmission routes, nonlinear timing, or factors not fully captured in structured data. The role of AI will be a particularly important adjunct for any health systems that implement WGS surveillance in multiple hospitals.

In conclusion, the EDS-HAT AI offers a significant advancement in the accuracy of outbreak investigation. By automating much of the data analysis, it reduces the burden on IP&C teams and enhances their ability to identify potential transmission routes that might be missed by traditional methods. Despite the challenges and limitations identified, our experience demonstrates the feasibility and benefits of such a system. Future work should focus on refining the algorithm, expanding its application across diverse healthcare settings, and continuing to address data integration and interoperability challenges.
